# Proceedings of the Third Annual Deep Brain Stimulation Think Tank: A Review of Emerging Issues and Technologies

**DOI:** 10.3389/fnins.2016.00119

**Published:** 2016-04-06

**Authors:** P. Justin Rossi, Aysegul Gunduz, Jack Judy, Linda Wilson, Andre Machado, James J. Giordano, W. Jeff Elias, Marvin A. Rossi, Christopher L. Butson, Michael D. Fox, Cameron C. McIntyre, Nader Pouratian, Nicole C. Swann, Coralie de Hemptinne, Robert E. Gross, Howard J. Chizeck, Michele Tagliati, Andres M. Lozano, Wayne Goodman, Jean-Philippe Langevin, Ron L. Alterman, Umer Akbar, Greg A. Gerhardt, Warren M. Grill, Mark Hallett, Todd Herrington, Jeffrey Herron, Craig van Horne, Brian H. Kopell, Anthony E. Lang, Codrin Lungu, Daniel Martinez-Ramirez, Alon Y. Mogilner, Rene Molina, Enrico Opri, Kevin J. Otto, Karim G. Oweiss, Yagna Pathak, Aparna Shukla, Jonathan Shute, Sameer A. Sheth, Ludy C. Shih, G. Karl Steinke, Alexander I. Tröster, Nora Vanegas, Kareem A. Zaghloul, Leopoldo Cendejas-Zaragoza, Leonard Verhagen, Kelly D. Foote, Michael S. Okun

**Affiliations:** ^1^Department of Neuroscience, Center for Movement Disorders and Neurorestoration, University of FloridaGainesville, FL, USA; ^2^Formerly affiliated with the International Technology Roadmap for Semiconductors (ITRS)Washington, USA; ^3^Neurological Institute Cleveland ClinicCleveland, OH, USA; ^4^Neuroethics Studies Program, Department of Neurology, Georgetown University Medical CenterWashington, DC, USA; ^5^Neurological Surgery and Neurology, Stereotactic and Functional Neurosurgery, Department of Neurosurgery, University of Virginia Health Science CenterCharlottesville, VA, USA; ^6^Department of Neurology, Rush University Medical CenterChicago, IL, USA; ^7^Scientific Computing and Imaging Institute, University of UtahSalt Lake City, UT, USA; ^8^Beth Israel Deaconess Medical Center, Harvard Medical SchoolBoston, MA, USA; ^9^Department of Biomedical Engineering, School of Medicine, Case Western Reserve UniversityCleveland, OH, USA; ^10^Department of Neurosurgery, University of California, Los AngelesLos Angeles, CA, USA; ^11^University of California, San FranciscoSan Francisco, CA, USA; ^12^The Emory Clinic, Emory UniversityAtlanta, GA, USA; ^13^Department of Electrical Engineering, University of WashingtonSeattle, WA, USA; ^14^Movement Disorders Program, Department of Neurology, Cedars-Sinai Medical CenterLos Angeles, CA, USA; ^15^Department of Neurosurgery, University of TorontoToronto, ON, Canada; ^16^The Icahn School of Medicine at Mount SinaiNew York, NY, USA; ^17^Greater Los Angeles Veterans Affairs Healthcare SystemLos Angeles, CA, USA; ^18^Department of Neurology, Alpert Medical School, Brown UniversityProvidence, RI, USA; ^19^University of Kentucky Medical CenterLexington, KY, USA; ^20^Department of Biomedical Engineering, Duke UniversityDurham, NC, USA; ^21^National Institute of Neurological Disorders and Stroke, National Institutes of HealthBethesda, MD, USA; ^22^Massachusetts General Hospital, Harvard Medical SchoolBoston, MA, USA; ^23^Department of Neurosurgery-Center for Neuromodulation, NYU Langone Medical CenterNew York, NY, USA; ^24^Neurological Institute, Columbia University Medical CenterNew York, NY, USA; ^25^Boston Scientific NeuromodulationValencia, CA, USA; ^26^Department of Clinical Neuropsychology, Barrow Neurological InstitutePhoenix, AZ, USA

**Keywords:** deep brain stimulation, local field potentials, neuromodulation, closed-loop, electrodes

## Abstract

The proceedings of the 3rd Annual Deep Brain Stimulation Think Tank summarize the most contemporary clinical, electrophysiological, imaging, and computational work on DBS for the treatment of neurological and neuropsychiatric disease. Significant innovations of the past year are emphasized. The Think Tank's contributors represent a unique multidisciplinary ensemble of expert neurologists, neurosurgeons, neuropsychologists, psychiatrists, scientists, engineers, and members of industry. Presentations and discussions covered a broad range of topics, including policy and advocacy considerations for the future of DBS, connectomic approaches to DBS targeting, developments in electrophysiology and related strides toward responsive DBS systems, and recent developments in sensor and device technologies.

## Introduction

The Third Annual Deep Brain Stimulation (DBS) Think Tank convened at the University of Florida's Research and Academic Center in Orlando, FL, on March 18-20, 2015. This report provides a summary of the conference sessions, which addressed the most current research, clinical, ethical and policy work on DBS for the treatment of neurological and psychiatric disease. DBS research and its clinical translation incur wide ranging, complex issues that necessitate ongoing frank discourse and exchange of ideas among the multi-disciplinary group of neurologists, neurosurgeons, neuropsychologists, psychiatrists, scientists, engineers, and ethicists developing and engaging DBS in research and clinical practice. The DBS Think Tank aims to provide an annual forum where contemporary issues, innovations, and challenges of the research and use of DBS are shared, discussed, and debated. Presentations and discussions addressed policy and advocacy considerations for the continued advancement of DBS, connectomic approaches to DBS targeting, developments in electrophysiology and related progress in responsive DBS systems, and recent innovation in sensor- and stimulation-device technologies.

The field continues to advance at an impressive pace. Our hope is that this meeting promotes awareness among stakeholders in DBS of currently unresolved and newly emerging issues, so as to ultimately strengthen the field and better serve patients. As in previous years, the meeting was conducted in a “think tank” style; speakers presented analyses of critical issues to foster dialog in subsequent discussions. The nature of this think tank format implies that this is not an evidence-based overview of developments in DBS; rather, it is a report of on-going developments that have been advancing this dynamic field and discussion of obstacles hindering further advancement and potential solutions. This summary includes key points of both the presentations and the follow-up discussions.

## Policy and advocacy for the future of DBS

### Viability of a DBS industry roadmap and consortium

An industry roadmap process, organization, success factors, and typical and expected outcomes were discussed, using the semiconductor technology roadmap as a prior example of successful effort (Spencer and Seidel, 1995; Schaller, 2001). Industry roadmaps provide a dynamic and evolving collaborative technology management process for determining critical needs and drivers, identifying technology and manufacturing targets, and assessing and modeling potential solutions to focus an industry community. They can also provide direction toward consensus-based resolution of needs and problems within specific timeframes (Rathore, 2009; Cartreine et al., 2010; Qattan et al., 2012; Finch et al., 2014). Such “road mapping” has existed within corporations and organizations for decades. Industry-wide roadmaps are versions of the corporate process that can effectively be used to identify gaps in solutions for common precompetitive challenges; suggest methods and programs to resolve those gaps, and address lead-time issues by indicating timeframes of opportunities for facilities, materials, and equipment development within a supply chain community (Garcia and Bray, 1997). Implementation can occur within organizations in the competitive space, thereby contributing to the growth of industry, by generating positive outcomes inclusive of strategic and tactical partnerships throughout the industry.

DBS is being used to mitigate signs and symptoms of an increasing range of neuropsychiatric disorders. In this way, DBS has attained considerable success in treating a greater number of patients, and in turn, fostered increased public awareness, receptivity and demand for this technology.

**Figure 1:**
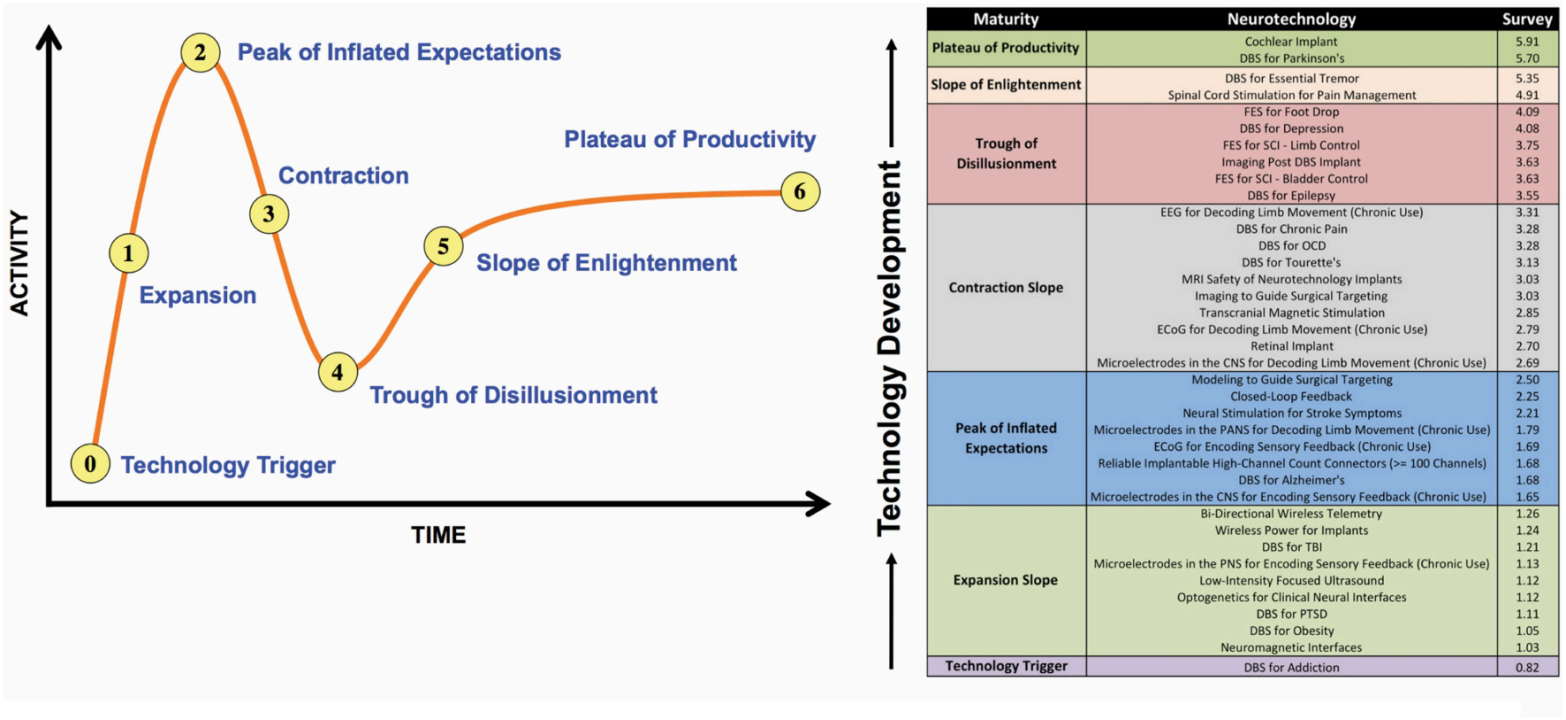
**(A)** A conceptual model of stages in technology development. **(B)** Results of survey evaluating participants' perceptions of neurotechnology development. Participants in the Think Tank were asked to submit examples of current or emerging neurotechnologies. A list was subsequently compiled and participants were asked to indicate where they believed each of the items ranked in terms of the six stages shown in **(B)**. Survey responses were averaged and the item was subsequently placed in the category corresponding to the nearest whole number.

The investment and success of DBS has fortified the viability of key technological, commercial, and clinical aspects of neurotechnology and are contributing to the continued expansion, development, and success of the field of neurotechnology in general and the use of DBS in medical practice in particular. As a result, there is substantial energy and investment to broaden the applications of DBS and to increase the capability and complexity of DBS systems. The question of whether and how the DBS industry would be best served by a technology roadmap and/or consortium may reflect the nature and extent of the common challenges presently impeding technology advancement and deployment. The field is laden with numerous issues, including increasing system complexity (e.g., increasing interfaces and channel count, telemetry bandwidth, recording and stimulation capability, etc.), variable biocompatibility, packaging challenges (i.e., demands for smaller size units with greater power efficiency and battery capacity), a proliferation of potential brain targets and indications, and these are reflected in—and foster—increasing regulatory requirements. Figure 1 presents an overview of expert perceptions of the state of maturity of DBS relative to other neurotechnologies, solicited from participants at the think tank in an anonymous poll.

Implementation of industry-wide standards can evoke both positive and negative effects for different stakeholders. For example, on one hand growing mandated regulatory standards can result in longer development times and higher development costs. On the other hand, such standards can fortify the integrity, efficacy, and safety of DBS technology in use, particularly now as the applications of DBS are expanding tremendously. In addition, the use of standards could actually lower barriers to market entry as certain components of DBS systems become more common commodities (e.g., the implanted pulse generator, wireless telemetry). If regulatory processes at the Food and Drug Administration (FDA) related to DBS systems evolve to approve key target-agnostic DBS system blocks or components, rather than entire systems designed for specific anatomical targets, it could significantly increase the rate of neurotechnology innovation. Specifically, such new regulatory processes would support the efforts of smaller companies, with novel algorithmic, anatomical target, and neural-interface concepts, to more quickly deliver solutions to an increasing diversity of patient populations, including those that are smaller and thus less economically appealing to the existing medical-design manufacturers. More attention is required to assess the potential viability of industry-wide roadmaps and consortia to resolve these issues.

### Policy to support physician initiated research and innovation

Physician investigator-initiated research (IIR) is generally regarded as conferring considerable advantages to DBS research compared to industry-sponsored studies. Physicians are more likely than industry to sponsor research focused on orphan and small disease populations, and increasing IIR in DBS would diversify and broaden ideas focusing upon the current challenges—and opportunities—in the field (Rossi P. J. et al., 2014). Moreover, as a group, physician researchers also have a longer time horizon for assessing outcomes and adding knowledge than most industry sponsors, which can lead to different and uniquely valuable types of studies. However, anecdotal evidence from physician researchers performing DBS trials suggests that significant regulatory burdens are slowing the pace of IIR research, and could be discouraging physicians from participating in such efforts (Rossi P. J. et al., 2014).

A case study providing an overview of the timeline and resources required to meet regulatory requirements for a DBS clinical trial was presented (Kelly et al., 2014). Financial costs for FDA-compliant data management were estimated at $100,000 USD for a 10 patient pilot study. Appropriate data management is critically important for both enabling maximum use of any and all information, and for protecting the privacy of patients and the integrity of the research. However, the costs of FDA-compliant data management substantially reduced funds available for performing the study. Regulatory consultants and support staff to interface with the FDA and to insure that all necessary requirements for the investigational device exemption (IDE) were met also contributed to overall regulatory costs, which in the case study were ~$75,000 per year. It was noted that such costs are prohibitive to many “stand-alone” investigators without federal funding or institutional resource sharing.

While industry-sponsorship of a study is possible for certain indications, such support is unlikely for research involving orphan disease populations that represent a small market share of potential consumers. Time costs were also significant; in the case study presented, it took three (3) years from approval of the NIH funding to the time that the first patient was enrolled in the trial. Time costs to the investigator also include dedicated efforts preparing regulatory paperwork. For example, it was estimated that the amount of hours required to prepare documentation for submission of an IDE was equivalent to the time required to prepare three (3) NIH R-01 grant proposals.

Even given these barriers to physician-initiated DBS research, it was emphasized that physicians are not seeking “less regulation” but instead are calling for regulatory reform. In this light, it was recommended that practices such as data review, process auditing, and unannounced site visits would be welcomed and could be increased, while processes related to documentation could be streamlined. Specifically, it was recommended that a FDA-approved template for DBS-related IDEs could guide investigators to more efficiently dedicate time and resources to the components definitively required for this type of research. In addition, it was recommended that the right of reference (RoR) process be reformed. Currently, RoR requires that industry partners approve studies that use their devices; this approval is a means of protecting commercial intellectual property and corporate assets and providing protection from litigation. It was suggested that this process could be reformed by indemnifying companies from the consequences of an investigator's FDA-approved off-label use of corporate intellectual property. This reform would center responsibility for the scope and conduct of research upon the physician, rather than the industrial sponsor. In turn, this would enable physicians' control over clinical studies and, at the same time, incur benefits to industry by no longer holding industry partners accountable for reviewing (and ultimately deciding upon) research proposals. Still, this reform may not resolve device manufacturers' concerns about a potential loss of confidence in their brand should a poor outcome occur.

**Figure 2:**
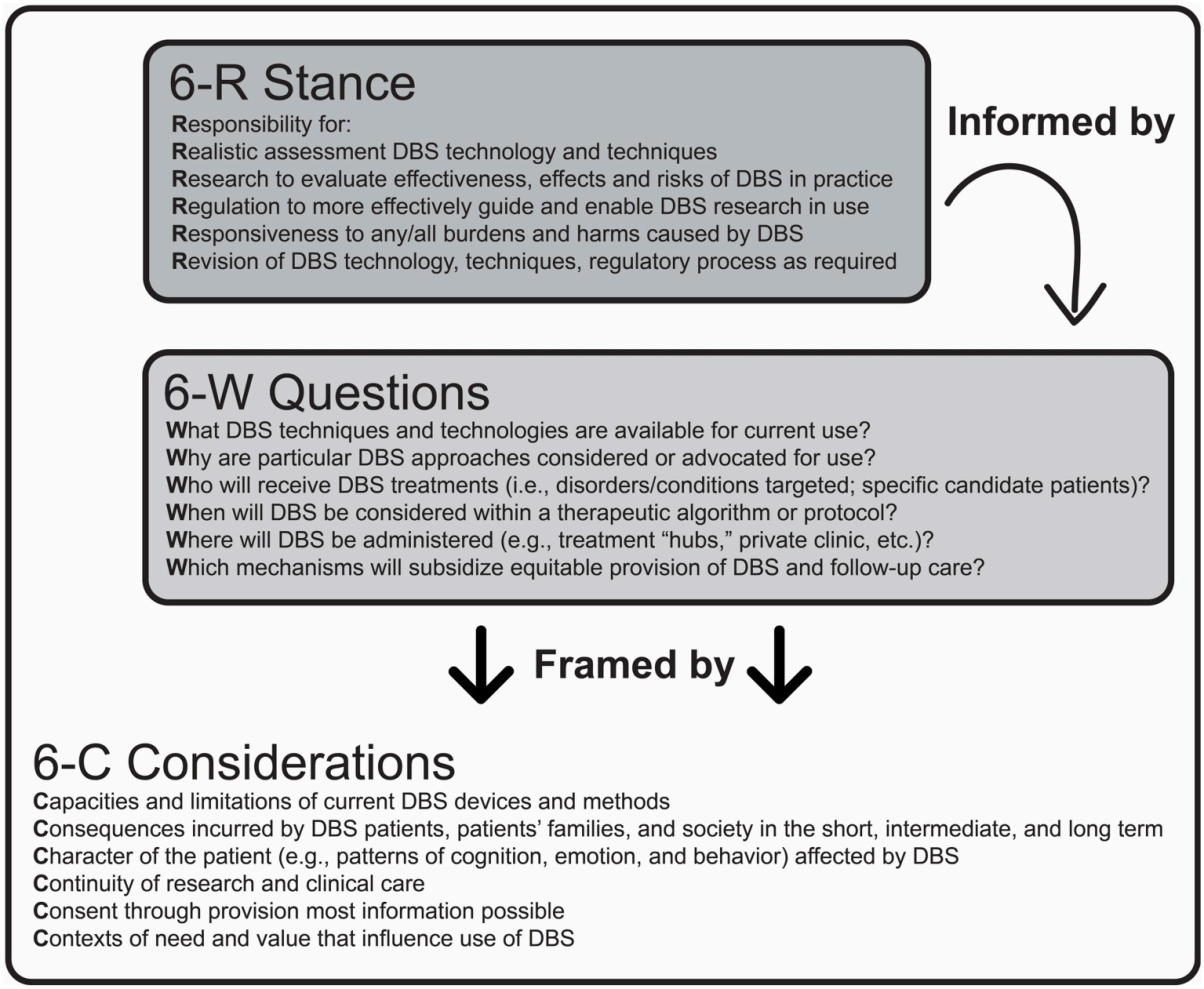
**Overview of the “6-R, 6-W, 6-C” model for the ethical development of DBS technology**.

The DBS field is becoming increasingly competitive, with potentially sizeable prizes, and sizeable risks. Achieving a leftward shift in the time course for IIR will require, attention to the benefits and burdens incurred in biomedical, ethical, and legal domains. Toward this end, a multi-step paradigm for comprehensively addressing critical issues and, importantly, guide forward progress in DBS research and its clinical translation was described and recommended. First, an overall “6-R” stance was advocated, which encourages responsibility for: assessment of capabilities and limitations of DBS in treatment of particular neuropsychiatric conditions, research to evaluate DBS effects in practice, regulation, responsivity to incurred burdens and harms and revision of DBS technology and techniques, and regulatory process, as necessary. Meeting these responsibilities invokes a “6-W” set of questions that can be used to define the parameters of use, and “6-Cs” that must be addressed in order to establish ethical probity in use (Giordano, 2015). Details of the “6-R, 6-W, 6-C” model for the ethical development of DBS technology are presented in Figure 2.

While the need for continuity of care for patients involved in experimental DBS interventions is clear from ethical and clinical perspectives, the actual provision of such longitudinal care has proven to be challenging in practice (Rossi P. et al., 2014). One vexing recurrent issue is that insurance providers occasionally decline reimbursing costs for off-label DBS, despite granting “pre-approval” in a peer-to-peer review process with the insurance company's medical directors.

Given the increasing diversity of DBS approaches, cumulative data aggregation, sharing, assimilation and synthesis will be increasingly important to the iterative assessment and improvement of the field. Toward these ends recommendation was made to establish a common database for DBS research and clinical outcomes, although the question was posed how—and through which entity or institution—such a common database would be established, hosted and curated (Giordano, 2012, 2014). Modification of extant systems, and development of new information management frameworks will be required to collect and integrate, support and sustain the wide distribution of many types and levels of data. Such approaches should: establish a common data format, optimize harvesting, aggregation and synthesis, establish checking systems to assess and characterize the type and quality of data, maximize accessibility and ensure the source of data, and enable retraction of data that are inaccurate or in need of revision for currency. In light of the increasing number and internationality of IIR DBS studies, it will also be important to address issues and questions of intellectual property and proprietary use (Brindley and Giordano, 2014). To fortify IP, provenance, attribution (and relative indemnities), and data use and sharing agreements will need to be implemented to achieve a dynamic repository that supports the range of intended uses for these data (clinical care, training, and research).

#### Highlights

1. The DBS industry may be served by a roadmap and/or consortium to address common challenges that are impeding novel technology development and deployment at present and in the near future.
2. Valuable investigator initiated research (IIR) could be strengthened by regulatory reform emphasizing data review, process auditing, and unannounced site visits while streamlining processes related to documentation.
3. FDA-approved template(s) for DBS-related investigational device exemptions (IDEs) could guide investigators to more efficiently dedicate time and resources.
4. There is an urgent need to establish databases for DBS research-related purposes.
5. Continuity of care concerns for patients involved in investigational DBS procedures must be considered.

## Innovative techniques and technologies in DBS

### Functional connectivity tools to guide stimulation for epilepsy

A critical step toward optimizing direct modulation of refractory focal-onset epilepsy is to effectively interface depth electrodes with complex epileptogenic brain circuits. Some novel approaches are currently being exploited to achieve this goal.

It was recently shown that a correlation-based measure of functional connectivity could be used to identify epileptogenic zones from intracranial stereoencephalography (SEEG) signals and that this information can be used to predict the outcome of lobectomy in intractable temporal lobe epilepsy (Antony et al., 2013). Indeed, patients with weakly connected, homogenous networks responded less favorably to temporal lobectomy. These findings suggest the value of such SEEG-based functional connectivity modeling in predicting the outcomes of depth electrode placement for epilepsy (Gonzalez-Martinez et al., 2013).

In addition, the FDA recently approved a depth electrode system as an adjunctive therapy for individuals with refractory focal-onset epilepsy with two epileptogenic sources. A novel pre-implant depth electrode placement planning system has been shown to enable the propagation of therapeutic current to communicating distant epileptogenic sources. The pre-implantation planning process consisted of several components: (1) structural magnetic resonance imaging (MRI) and diffusion tensor imaging (DTI) datasets; (2) computation of the induced electric potential surrounding the electrode contacts using a finite element method; (3) analysis of the effect of the electric field-dependent FA (fractional anisotropy) model on depolarizing axon bundles as identified by high-resolution DTI; and (4) predicting distant cortical activation by strategically placing the FA volume seeds to create a modulated circuit tractography (MCT) map. The pre-implant MCT map was then used as a targeting template for placing up to two depth leads intra-operatively. This planning system was validated via subtraction activated SPECT (SAS), which is a perfusion imaging technique that captures stimulation induced transient changes in cerebral blood flow. SAS was utilized post-implantation to validate *in vivo*, the maximal extent of epileptogenic regions influenced by stimulation therapy.

### Functional connectivity tools enable personalized DBS

The effect of focal brain stimulation is not limited to the region targeted and a DBS current can propagate through anatomical connections to influence distributed neural networks in the brain. Emerging techniques that can help DBS practitioners visualize these networks are likely to prove valuable for understanding and guiding brain stimulation. One imaging technique particularly well suited to visualizing brain networks is resting state functional connectivity magnetic resonance imaging (fcMRI) (Fox and Raichle, 2007). This technique has already been demonstrated to (1) identify thalamic DBS targets based on connectivity to brain regions implicated in tremor (Anderson et al., 2011), (2) link invasive and non-invasive brain stimulation sites across 14 different neurological and psychiatric diseases (Fox et al., 2014), and (3) be safely applied in patients implanted with DBS electrodes using special low-energy MRI sequences (Kahan et al., 2014).

Another method that has emerged with considerable promise is patient-specific tractography-activation models (TAMs), which can enable the identification and visualization of white matter pathways activated by brain stimulation. TAMs essentially predict action potential generation in specific pathways. They combine anatomical imaging data, probabilistic tractography from the brain region surrounding the implanted DBS electrode, models of the electrical fields generated by DBS parameter settings, and cable models of axons (Lujan et al., 2013). TAM may lead to improved personalized surgical targeting and stimulation parameter selection. TAM may also facilitate identification of new DBS targets (Downes and Pouratian, 2014), and a deeper understanding of the mechanisms underlying both the therapeutic and off-target effects of DBS (Riva-Posse et al., 2014; Sweet et al., 2014).

Harnessing advances in neuroimaging techniques may also play a role in re-evaluating conventional thinking in the field. For example, with probabilistic diffusion tensor tractography, the four anatomical targets for DBS in cluster headache described as hypothalamic in literature were shown to be localized to the midbrain tegmentum posterior to the hypothalamus. Importantly, tractography also revealed common tracts across these targets, which included projections to the ipsilateral hypothalamus, reticular formation, and cerebellum (Clelland et al., 2014). Collectively, these results can motivate a shift from stimulation of specific brain targets to stimulation of specific brain networks.

### Investigating lead placement variability

There can be considerable variability in DBS outcomes, and some clinical trials have failed recently because of profound variability in response rate across the patient cohort. This is evident even in successful clinical trials. For example, in one of the larger DBS trials in PD the standard deviation in clinical outcome scores was roughly equal to the effect size (Deuschl et al., 2006). Over the past 10 years computational models have been used to characterize potential sources of variance in the way DBS is applied (Grill et al., 2004; Johnson and McIntyre, 2008; Dorval et al., 2009, 2010; Santaniello et al., 2011). In general, these studies attempted to characterize how and where stimulation was applied in each patient. With regard to the latter, one critical element for both surgical planning and population research has been co-registration of pre-operative patient MRI to a brain atlas. This is performed prior to surgery to permit indirect targeting of nuclei that have poor contrast on conventional imaging, and it is performed after surgery so that regions of activation for each patient can be expressed in a probabilistic atlas of outcomes (Butson et al., 2011). During this process it has been observed that lead locations often vary within and among surgical sites. This observation has led to questions about errors that could be introduced during atlas registration, and has motivated an evaluation of the accuracy of this registration process. The most important finding from this evaluation was that the observed variability in lead location cannot be attributed to errors introduced during atlas registration. In fact, three different registration algorithms yielded virtually the same results. This information supports the suggestion that the neuromodulation community could benefit from wider adoption and acceptance of open source registration algorithms, several of which have been rigorously developed and tested.

### Temporal pattern of stimulation as a new dimension of therapeutic innovation

Although DBS is an established therapy for the treatment of movement disorders, debate persists about the mechanisms by which high frequency stimulation reduces symptoms. This probably results in a failure to achieve full optimization of the therapy with maximal benefits and minimal side effects. Thus, an improved understanding of therapeutic mechanisms will be important to enable further innovations in DBS technique and technology.

The cellular effects of DBS on neurons of the central nervous system include simultaneous inhibition of the cell body and activation of the axon (McIntyre et al., 2004). This finding motivated the “informational lesion” hypothesis positing that DBS masks pathological oscillatory activity by normalizing the activity of neurons within the stimulated nucleus. The striking parallel between the frequency-dependent effects of DBS on regularizing the activity of model neurons and the clinical effects on symptoms provides strong correlational evidence for this hypothesis (Grill et al., 2004). The informational lesion hypothesis has been tested in several recent experiments (Zimnik et al., 2015). The changes in representation of kinematic information in the firing patterns of neurons of the globus pallidus and thalamus that occurred during DBS indicate that effective DBS produces at least a partial disruption of neural information (Agnesi et al., 2013).

In contrast, a more recent study concluded that DBS does not disrupt information transmission in the basal ganglia (Zimnik et al., 2015). However, the currents used in this study were orders of magnitude smaller than those required for positive effects on symptoms, and therefore the effects of DBS on neuronal activity were substantially underestimated. Using a highly innovative paradigm to render a temporary direct connection to the brain lead during surgical replacement of the implantable pulse generator (IPG) enabled another test of this hypothesis (Swan et al., 2014). Random patterns of subthalamic nucleus DBS, even when delivered at a high average frequency (130 Hz), were not effective in relieving bradykinesia in patients with Parkinson's disease. These findings reinforced the potential importance of regularization (rather than complete disruption) of neuronal activity for the effectiveness of DBS (Dorval et al., 2010).

The finding that the effects of DBS were dependent on the temporal pattern of stimulation, in addition to the frequency of stimulation, inspired the design and testing of novel temporal patterns of DBS. Patterns were developed that treat the symptoms of Parkinson's disease (PD) more effectively than conventional regularly patterned DBS (Brocker et al., 2013). or alternatively enabled equivalent treatment of symptoms but with a substantial reduction in the required energy. This latter innovation is an important consideration for the size, recharge frequency, and battery life of implanted pulse generators. Collectively, the results demonstrated the utility of an entirely new dimension of neural stimulation parameters—the timing between stimulation pulses—to increase the efficacy and efficiency of stimulation.

### Advancements in lead design

**Figure 3:**
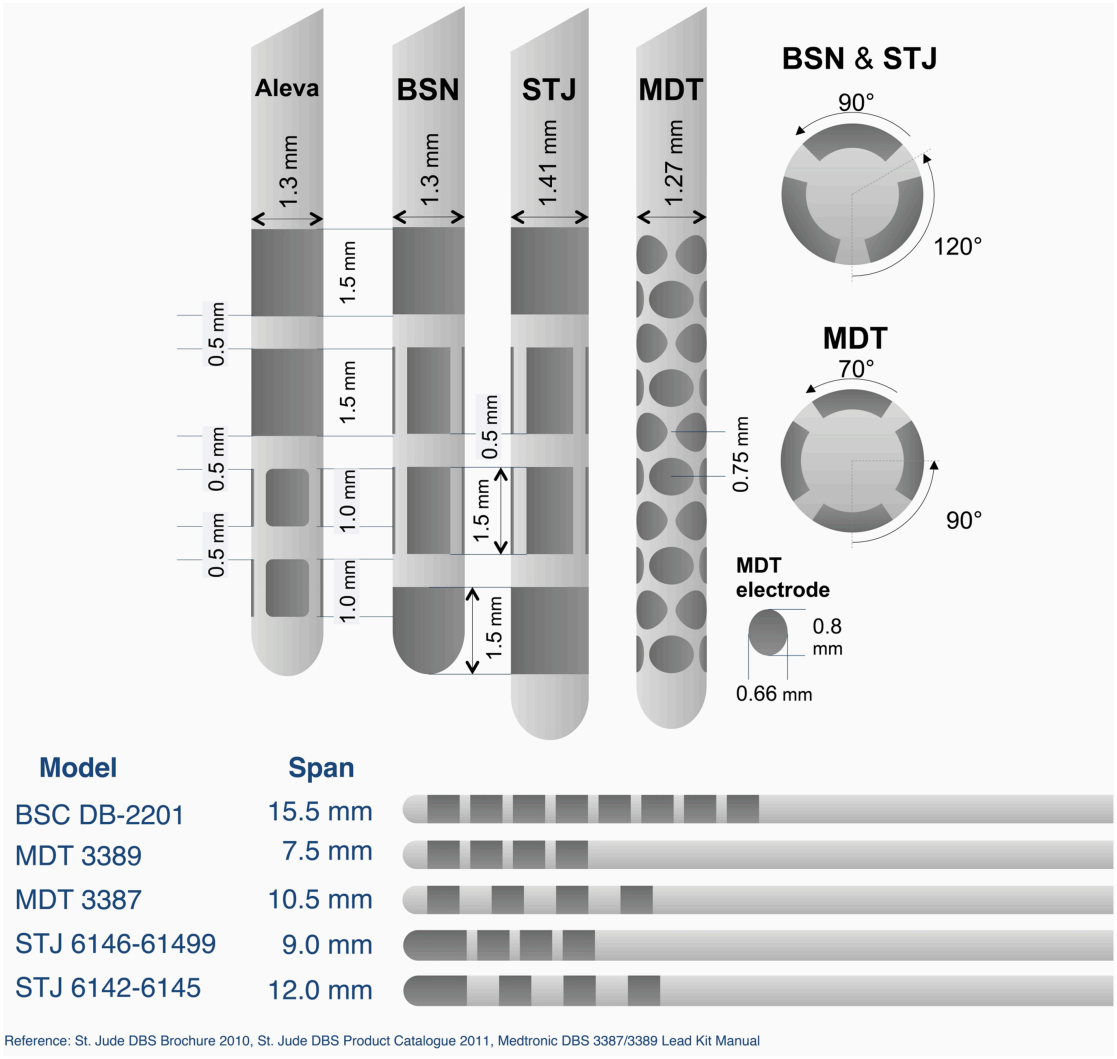
**Comparison of emerging DBS electrode lead technology**. BSN, Boston Scientific Neuromodulation; STJ, St. Jude Medical; MDT, Medtronic. Reference: St. Jude DBS Brochure 2010, St. Jude DBS Product Catalog 2011, Medtronic DBS 3387/3389 Lead Kit Manual.

Emerging DBS device technology will enable controlling of stimulation fields. Three novel lead designs—all with uniquely engineered approaches to achieving this objective—were presented. Two of the presented leads feature electrodes segmented radially about the lead, in contrast with existing leads that are segmented only along the long axis of the lead. The third lead featured an extended span, and brings with it a new kind of current control to DBS. These new leads will allow the stimulating currents to be programmed in order to preferentially stimulate therapeutic targets and avoid stimulating areas prone to side effects. The importance of tuning and directing the stimulating field is based on the observation that electrodes are occasionally sub-optimally placed (Okun et al., 2005) and sometimes quite far from the intended target (Ellis et al., 2008). All three of the lead designs are currently being evaluated in various clinical trials (Contarino et al., 2014; Pollo et al., 2014; Vitek and Starr, 2015). The use of directional electrodes can improve DBS outcomes when electrodes are sub-optimally placed. Preliminary data presented at the Think Tank indicated that targeted stimulation with these approaches is promising, although the long-term benefits of the “directional” leads remain to be demonstrated, particularly in a chronic study. Figure 3 provides a detailed comparison of these new lead designs.

One lead under investigation (Medtronic-Sapiens) possesses an advanced multiplexer unit that supports a total of 40 electrodes and a span of 7.41 mm. With 10 rows of 4 electrodes per row, and alternating rows offset by 45°, 8 radial electrode directions are possible. Stimulation can be further shaped and aimed radially by choosing various combinations of active electrodes and splitting the current between them; however, details are not yet public regarding how electrodes may be combined and programmed. Additionally, recording of local field potentials (LFPs) is possible from each of the 40 electrodes—potentially yielding spatiotemporal information on pathologic neuronal activity. Preliminary intraoperative testing of this system suggests that it may be possible to utilize intraoperative LFP recordings to assess the effect of stimulation in different electrode combinations and current settings on pathological subthalamic electrical activity (Bour et al., 2015). This field shaping capability can possibly avoid stimulation of unwanted regions and enhance engagement of target areas (Barbe et al., 2014).

Another recently tested lead (Aleva) has a total of eight electrodes (span, 5.5 mm): two are the traditional ring electrodes and the remaining two rings are divided into three segments each, allowing for directional current delivery through each segment (Chase, 2014; Pollo et al., 2014). The electrode corners are rounded so as to avoid “hot spots.” Clinical data suggest that this directional lead, when tested in the acute setting in either the subthalamic nucleus (STN) or the ventralis intermedius (VIM) thalamus, can improve the therapeutic index, i.e., enlarge the window between therapeutic effect and adverse effects, and also may possibly use less current to achieve the same therapeutic benefits (Chase, 2014; Pollo et al., 2014).

Another lead (Boston Scientific DB-2201; currently undergoing clinical evaluation in the United States) also possesses eight electrodes (15.5 mm span), current on each of which can be precisely controlled, as the IPG is capable of current steering. The aim of this design, also referred to as Multiple Independent Current Control (MICC), is to achieve more precise targeting of stimulation by enhancing control of the therapeutic electric field. As the IPG associated with this lead is a current control design, it should theoretically enable more stable stimulation. By reducing variability in impedance and permitting the effective use of lower pulse widths, it should also be possible to expand the therapeutic window of effective current amplitudes. Results of a European trial have shown clinical outcomes comparable to existing leads, and during the trial over 70% of programmers utilized the current steering feature (although motivations for this use have not yet been evaluated; Timmermann et al., 2015). The system also has cordless recharging, and the rechargeable IPG utilizes cero volt technology, which helps prevent substantial loss in battery capacity following frequent and/or full discharges. Future capabilities that could be co-deployed with this lead were discussed, such as computer-guided distribution of current across contacts for an optimized or informed programming.

### Non-DBS technology impacting the field: focused ultrasound

In addition to addressing important improvements in DBS system technology, the Think Tank sought to identify “non-DBS” approaches that could impact the DBS field. In this regard, the use of transcranial high intensity focused ultrasound (HIFU) for treatment of movement disorders was reviewed (Dallapiazza et al., 2014). While effective, stereotactic lesioning of the brain for the treatment of movement disorders has been largely abandoned with the development of DBS (Dallapiazza et al., 2015). However, progress in transcranial MR-guided focused ultrasound technology has renewed an interest in stereotactic lesioning mainly because of the potential for continuous MRI-guidance of an “incisionless” thalamotomy (Wang et al., 2015). Three pilot studies at different institutions have demonstrated significant improvements in hand tremor in patients with severe essential tremor following focused ultrasound thalamotomy (Elias et al., 2013; Wintermark et al., 2014a,b). These studies have suggested functional improvements in activities, disabilities, and quality of life with minimal morbidity. Furthermore, transcranial ultrasound at low intensities can be used to manipulate deep brain circuitry through non-invasive brain mapping prior to lesioning. Clinical trials of HIFU for mapping neural circuitry and treating in essential tremor and PD are currently ongoing.

Proponents of HIFU further highlight that an incision and burr hole are not required to perform the procedure, offering a “lower-cost, less invasive” alternative to DBS that eliminates both the risks of penetrating the brain and the inconvenience and costs imposed by implanted hardware (Lipsman et al., 2013). Dissenters believe this to be an oversimplification. Unlike DBS surgery, HIFU *requires* the head to be completely shaved; patients must remain awake during the procedure and must lie flat within the MRI scanner for a few hours while the target is localized. The MRI environment, while offering the potential for procedural monitoring, can be difficult to work in and some patients cannot tolerate these image-guided procedures. In comparison, DBS targeting scans are obtained in just a few minutes and patients are positioned more comfortably during surgery. Most importantly, HIFU is an ablative and irreversible lesion, and can result in adverse effects especially when used bilaterally.

Direct comparisons of traditional (non-HIFU) thalamotomy and thalamic DBS have already been performed (Tasker, 1998; Schuurman et al., 2000; Pahwa et al., 2001). Three studies, conducted at reputable centers in the U.S., Canada, and Europe have all reached a similar conclusion. While initial tremor control was comparable for the two interventions, thalamic DBS was safer than thalamotomy, causing fewer neurological complications and reducing the need for re-operation in the event of tremor recurrence. Indeed, one member of the University of Virginia HIFU cohort suffered permanent dysesthesia (Elias et al., 2013), and a follow-up study from that group suggests that tremor control may lessen over time as the lesion shrinks in size (Wintermark et al., 2014a). However, it is worth noting that ET can also become refractory to or tolerant of DBS despite increasing currents (Favilla et al., 2012). Clearly, there is a subset of patients who will subjectively prefer HIFU to DBS; however, it remains unknown whether HIFU ablation is objectively better or cheaper than DBS of the thalamus or any other target. Moreover, there are concerns over limitations of this technology including the need for bilateral procedures, the safety profile, and the lack of programmability.

#### Highlights

1. SEEG-based functional connectivity modeling is helping to predict outcomes of depth electrode placement for epilepsy (Gonzalez-Martinez et al., 2013).
2. Connectivity-based approaches, both functional and structural, suggest that targeting brain networks rather than individual brain sites may improve and personalize DBS.
3. Modifying the temporal pattern of DBS stimulation may offer a new dimension to the therapy.
4. Emerging DBS lead designs incorporating radially segmented electrodes and systems incorporating current steering will enable greater specificity in brain circuit targeting and will improve the benefit/side effect ratio of DBS therapy.
5. Stereotactic ablation with high frequency ultrasound is a less invasive procedure compared to DBS, but carries considerable limitations, including irreversibility and unilaterality.

## Closing the loop with local field potentials

### Local field potentials provide insight into neuropsychiatric disorders

DBS has shown promise as a therapy for neuropsychiatric disorders; however, advances have been hindered by relatively poor understanding of the neural networks involved in the pathophysiology of these conditions. To address this gap, an ongoing study by researchers at University of California San Francisco is investigating the mechanisms underlying depression and anxiety in PD patients undergoing awake DBS surgery.

This population of patients is ideal for studying the circuits involved in these depressive and anxious symptoms because they frequently co-occur in varying degrees of severity in Parkinson's disease (Gallagher and Schrag, 2012). These symptoms may be modulated by both DBS and dopamine replacement therapies (Tan, 2012; Storch et al., 2013). High spatial resolution recordings of neuronal activity are performed intraoperatively within brain structures that have been shown to be involved in emotion regulation and cognitive control. Field potentials are recorded from the DBS lead (containing 4 electrode contacts) implanted in the basal ganglia (either the subthalamic nucleus or globus pallidus) and from an additional subdural electrode (containing 28 contacts, spaced 2 mm apart) placed over the prefrontal cortex. Cortical areas targeted include the dorsolateral/medial prefrontal cortex, the orbitofrontal cortex, and the ventrolateral prefrontal cortex. Recordings are performed while patients rest or perform tasks that engage networks involved in emotion regulation and cognitive control. In addition, cortical and subcortical stimulation are used to modulate mood. To identify correlations between neural physiology and symptom severity, depression, mood, and anxiety are characterized both before the surgery using a variety of validated scales, and during the surgery using visual analog mood scales.

Preliminary results of these experiments in the prefrontal cortex suggest that, similar to what has been observed in the motor cortex (Shimamoto et al., 2013; Yang et al., 2014; de Hemptinne et al., 2015), the prefrontal cortex is dominated by beta oscillations that can be coupled to broadband gamma activity (Hammond et al., 2007). It was found that the magnitude of beta rhythm and phase-amplitude coupling varies between patients. In addition, in few patients, it was shown that stimulation delivered to the deepest contact of the DBS lead can induce anxiety, and this effect on mood is associated with a decrease in beta activity and an increase in broadband gamma. Although preliminary, these findings suggest that both the beta oscillations and broadband gamma activity might be relevant to psychiatric symptoms, and that the excessive synchronization observed in cortical-basal ganglia motor networks might also occur in cortical-basal ganglia networks involved in emotion and cognition.

### Long-term cortical and subcortical local field potentials (LFPs) in parkinson's disease

While DBS is an effective treatment for movement disorders such as PD there are several ways that it might be improved. The current approach is to deliver constant stimulation without adjusting the therapy or controlling for the patient's disease state, medication status, or side effects. A goal of advancing DBS therapy is to implement a “closed-loop” system where electrical signals from the patient's brain are used in real-time as feedback to customize stimulation delivery. Closing the loop can be used to reduce undesired side effects of stimulation and to extend battery life, as well as to improve stimulation effectiveness.

An important step in the development of closed-loop DBS for PD is the characterization of brain signals associated with the disease-relevant network. A recent on-going study aimed at identifying pathophysiological activity related to PD implanted five patients with novel devices capable of both stimulating and long-term recording and storing of LFP data. In this study, patients are implanted with a DBS electrode in the STN capable of both stimulation and sensing/recording electrical activity. In addition, 4-contact electrocorticography (ECoG) strip placed over the primary motor cortex (M1), which is used for sensing/recording only. Each patient is tested multiple times, both on and off medication, and on and off DBS. Preliminary results reveal that in most cases, medication is associated with a reduction in beta (13–30 Hz) power in the STN, while no consistent changes in beta power are recorded from M1. While these results support the importance of beta synchrony throughout basal ganglia-thalamo-cortical loops in PD, they also suggest that there is variability between patients, and that a closed loop signal may need to be optimized on a patient-by-patient basis, and/or that a combination of control signals may be needed.

In addition to identifying markers that are of relevance to hallmark PD symptoms, this study also aimed to assess adverse effects associated with dopaminergic therapy such as the dyskinesias. In two patients experiencing marked contralateral arm dyskinesia, a consistent and reproducible emergence of a narrow-band ~70 Hz increase in cortical power was observed. There was also increased coherence in the same frequency range between STN and M1. Similar patterns of high frequency, narrow-band activity has been previously observed in a rodent model of dyskinesia (Halje et al., 2012), and these may be useful for closed loop approaches.

### Long-term cortical and subcortical LFPs in tourette's syndrome

Tourette Syndrome (TS) is a paroxysmal neuropsychiatric disorder characterized by involuntary movements and/or vocal outbursts (i.e., - tics) typically preceded by a premonitory urge (Cheung et al., 2007; Kenney et al., 2008). DBS has been used to treat cases of severe and intractable TS (Almeida et al., 2015). It is estimated that ~120 TS patients worldwide have been treated with DBS since 1999, and almost 48 published studies report some degree of motor tic reduction (Schrock et al., 2015). While initial trials have been promising, the mechanisms subserving the effectiveness of DBS in reducing TS signs and symptoms have yet to be identified. Current models of TS hypothesize that thalamocortical-basal ganglia dysfunction is a key network underlying many TS symptoms. Inhibitory input from basal ganglia structures affecting the activity of key thalamic nuclei likely plays a role in patterns of motor behaviors. It may be that inhibition of basal ganglia structures leads to disinhibition of thalamic nuclei, which ultimately evokes initiation of tics (Perlmutter and Mink, 2006). Previous research has demonstrated potential biomarkers of tics (Maling et al., 2012; Bour et al., 2014). Building upon this work, the validity, reliability and relative predictive value of these biomarkers, and the development of an algorithm that can be used in the early detection of tics was presented.

In the presented study, two patients with severe, medication refractory TS were implanted with bilateral DBS devices. Depth leads were placed in the centromedian-parafascicular nucleus of the thalamus (CM-PF) and electrocorticography (ECOG) strips were placed over the precentral gyrus. Experiments consisted of separate interleaved trials in which patients were instructed to (1) tic freely, (2) suppress tics (baseline), and (3) execute volitional movements (shaking hands rapidly, opening and closing hands, raising arms up, and down, talking). Data were recorded intraoperatively and post-operatively. Intraoperative recordings demonstrated that both significantly more low (1–10 Hz) and high (30–100 Hz) frequency CM-PF activity was present during tics but not during volitional movements. A support vector machine (SVM)- based detector (Temko et al., 2011; Wissel et al., 2013) was constructed to investigate the relationship between this activity and tics during each post-operative visit (for a period of 6 months). Three types of tics were recorded including simple, complex, and long complex tics. Long complex tics were shown to be concurrent with a consistently detectable thalamocortical signature. Short complex tics were more difficult to detect than long complex tics, and simple tics were the most difficult to detect. Acute trials of closed loop stimulation using the human tic detector are currently underway.

### Local field potentials and depression

A confluence of information prompted exploration of the subcallosal cingulate gyrus (SC) as a DBS target for treatment resistant depression (TRD) (Lozano et al., 2008). Attempts to reduce TRD with SC DBS have shown clinical benefit: a recent study involving 10 patients showed significant response and remission rates following SC DBS (Holtzheimer et al., 2012). However, numerous other targets for TRD, including the nucleus accumbens, dorsolateral prefrontal cortex, and the lateral habenula, have been proposed and subsequently explored in clinical studies, with many of these showing at least some evidence of clinical benefit (Rosa and Lisanby, 2012). The question then arises if and how these targets may be related. The conceptualization of depression as a network disorder suggests that neuromodulation at the purported origin as well as at “nodes” of the network can be beneficial (Mayberg, 2009). TAMs of patients who underwent SC DBS for depression demonstrate significant differences between those who responded to therapy and those who did not (Riva-Posse et al., 2014). Specifically, it was shown that responders had greater tract coverage in critical regions.

Recent work has focused on using this information to improve DBS targeting. Prospective work is currently underway to utilize DTI pre-operatively to plan lead placement. This approach has been associated with an increase in the 6-month response rate from 41 to 76%. An important next stage of research will be to identify other markers that can confirm if a lead has been placed in a location that would elicit maximal benefit. On-going studies aimed at identifying physiological markers useful in evaluating lead placement and predicting treatment response were presented. These studies focused on intraoperative LFPs from the DBS electrodes in conjunction with intraoperative ECoG and electroencephalograhy (EEG). Previous studies have identified changes in alpha rhythm in depression; the alpha band frequency is increased in the left frontal lobe in depression (Saletu et al., 2010) while alpha is decreased in the right prefrontal cortex (PFC) (Bruder et al., 2001). Preliminary studies of intraoperative LFPs from both ECoG and depth electrodes after stimulation at “effective” contacts (determined by imaging) have demonstrated decreases in the alpha and beta frequencies in the left PFC, as well as a decrease in alpha and beta bands in the subgenual cingulate.

### Local field potentials and strides toward a closed-loop DBS

DBS systems currently deployed in the clinic are “open-loop” and do not take into account the potentially intermittent nature of symptoms. By detect the neurophysiologic correlates of symptoms such as tremor, we can determine not only when stimulation may be necessary but also estimate the intensity of stimulation needed. Stimulating only when necessary can increase the battery life of the implanted devices and reduce a patient's exposure to unintended effects.

A novel mobile, wireless platform for investigating closed-loop DBS applications in ambulatory patients was presented (Herron and Chizeck, 2014). The platform consisted of a set of body-worn sensors communicating wirelessly to a host application running on a smartphone or a personal computer. Taking advantage of movement data including inertial measurements, electromyography, and LFPs, these host applications are capable of performing digital signal processing and data fusion in order to make control decisions. These control decisions can include enabling or disabling stimulation or modifying individual stimulation parameters (voltage, pulse width, frequency) in response to changes in neurological symptoms (Herron et al., 2015). These control decisions are then sent wirelessly to an external receiver that then relays packets and control decisions to an implanted neurostimulator. This real-time command link to the implanted device has enabled the implementation of an integrated closed-loop DBS system.

This system confers several important benefits for both research and patient care. Currently, studies are underway to assess clinical performance of the system, and future studies are being planned which utilize the wealth of consistent, chronic data generated from the integrated system to investigate neurological movement disorder, particularly tremor.

#### Highlights

1. Chronic recording of FPs is permitting greater insight into multiple neuropsychiatric disorders.
2. LFP-based research holds promise for the identification of pathological brain signals that could serve as triggers for responsive stimulation.
3. Closed loop systems are being tested which could use a variety of signals in order to modulate therapy.

## Innovative targets for new indications

### Lateral habenula as a target for depression

In 2007, a non-human primate study provided the first electrophysiological evidence that the lateral habenula (LHb) played a role in the brain's reward system. The study demonstrated that reward was associated with suppression of inhibitory input from the LHb and subsequent activation of dopamine neurons, while the reverse was observed for non-rewarding trials (Matsumoto and Hikosaka, 2007). That same year, Alex Sartorius and Fritz Henn advanced the hypothesis of over activation of the habenula in major depressive episodes and argued that DBS of the lateral habenula could be beneficial for TRD (Sartorius and Henn, 2007). More recent studies in humans have corroborated the notion of LHb involvement in the reward system (Salas et al., 2010), and, interestingly, two case reports have since been published (2010, 2013) showing remission of major depression under DBS of the LHb (Sartorius et al., 2010; Kiening and Sartorius, 2013; Schneider et al., 2013). Early results from an on-going open-label trial of six patients undergoing habenular DBS for TRD were presented. The hypothesis that patients who were responsive to electroconvulsive therapy (ECT)—even if for a short duration—could be “better” candidates for habenular DBS was proposed, and is under investigation in the present study. As a DBS target, the LHb poses unique challenges for electrode implantation—among these is the proximity of the target to the third ventricle and the resultant motion artifact observed in imaging studies due to the pulsatile movement of the cerebro-spinal fluid (CSF). Programming-related adverse events observed to date were discussed. Upper extremity paresthesias occurred commonly, although patients mostly habituated to this and the effect was modifiable by gradually increasing the current. Oculomotor abnormalities were also observed which limited current dosing, and it was proposed that current steering could be useful in limiting this side effect.

### DBS of the basolateral nucleus of the amygdala for PTSD

Neuromodulation of the amygdala may prove beneficial in disease processes where symptoms arise from the aberrant assignment of an emotion to a specific event or context (Langevin, 2012). In post-traumatic stress disorder (PTSD), patients assign fear to benign situations, which may potentially lead to avoidance behavior. Several neuroimaging studies have demonstrated that the amygdalae of PTSD patients are metabolically overactive during symptomatic episodes (Etkin and Wager, 2007; Hughes and Shin, 2011). The level of activity within the amygdala correlates with the severity of the symptoms as measured by the clinician-administered PTSD scale (CAPS). An important study by Koenigs and colleagues showed that Vietnam veterans who suffered traumatic brain injury to the amygdala never developed PTSD (Koenigs et al., 2008). These results suggest that the amygdala plays a critical role in the production of PTSD symptoms. Focal interference of amygdala activity through DBS may improve PTSD. Although its mechanism is not fully understood, high frequency DBS is thought to functionally inactivate a specific, gray matter target. It was previously shown that DBS of the basolateral nucleus of the amygdala (BLn) reduced the behavior associated with PTSD in a rodent model (Langevin et al., 2010). A subsequent study in the same rodent model demonstrated that BLn DBS was superior to paroxetine—one of the drugs approved by the FDA to treat PTSD (Stidd et al., 2013). These results led to the recent development of a clinical trial to evaluate the feasibility and the safety of this technique in PTSD patients (Koek et al., 2014). An overview of the trial design was presented, as well as an overview of the first surgical subject implanted. In addition, the targeting technique and intra-operative microelectrode recording findings were described. Consideration of the patient's neuroanatomy is critical because of the wide variation in size and shape of the mesiotemporal structures.

### DBS for stroke

The concept of post-stroke neurostimulation does not focus on modulation of the area damaged by ischemia, rather it is intended to (1) augment the perilesional cortex or (2) modulate other areas whose connectivity has been disrupted by the stroke. Direct cortical stimulation as a means of enhancing excitability and plasticity has been investigated (Alonso-Alonso et al., 2007), but has failed to produce the intended benefits in clinical trials. One possible reason for the failure of cortical stimulation was the relationship between cortical axons and the source of stimulation (Manola et al., 2005a,b). In rodent models, axonal arrangement perpendicular to the cathode is predictable. However, in human brains, axonal arrangement is less predictable, and it is possible that axons were inhibited and excited in near equal proportions during cortical stimulation interventions and the net effect washed out. A novel approach to stroke therapy via DBS was discussed; the intention was to stimulate natural fiber pathways to the perilesional cortex in a way that mimics their native function. Preliminary results of this design in a rodent model were presented. Based on motor pathway fiber tracing by Dum and Strick, a cerebellar target (lateral cerebellar nucleus) was selected with the goal of modulating the dentatothalamocortical pathway (Dum and Strick, 2002; Machado and Baker, 2012). Stimulation of this target (particularly at 30 Hz) was shown to evoke cortical excitability. A follow-up study tested the effect of chronic 30 Hz DBS in this target on motor function in a rodent model of stroke (Machado et al., 2013). Animals in the stimulation group showed a significant improvement in motor function compared with post-ischemia baseline performance as well as in comparison with the non-stimulation group (Machado et al., 2013). Moreover, perilesional synaptic density testing showed that animals in the stimulation group had significantly greater numbers of perilesional synapses. Preliminary results of on-going studies of cytoarachitecture in these animals addressing whether these observations reflect neurogenesis in addition to synaptogenesis were also presented. Substantial discussion surrounded the topic of the appropriate level of pre-clinical evidence needed to justify early clinical translation studies.

#### Highlights

1. Emerging research suggests the viability of DBS as a treatment for new indications, including depression, PTSD, and stroke.
2. Research into novel targets for novel indications requires hypotheses based on animal models (where possible), well-designed clinical trials, and careful attention to potential off-target effects, patient selection, and targeting considerations.

## Conclusion

These proceedings represent the deliberations of the third Annual Deep Brain Stimulation Think Tank. The group addressed critical issues affecting the progress of the DBS field. These issues span multiple domains, including regulatory and ethical issues as well as study design. There are also important barriers to advance electrophysiology and system engineering. In discussing these challenges, participants in the Think Tank proposed and discussed possible solutions.

Scientific, clinical, and engineering advances that could transform the DBS field in the near future served as a primary focus of the Think Tank. The meeting focused on recent discoveries that may lead to transformative, not just incremental change in DBS therapy. Participants discussed the broad range of potential applications of the new knowledge, techniques, and technologies presented; they also discussed ways in which these advances could be fully exploited to rapidly advance to the next generation of DBS therapy. The future of DBS will depend heavily on building on these advances and on filling knowledge current gaps.

## Author contributions

All authors listed, have made substantial, direct and intellectual contribution to the work, and approved it for publication.

### Conflict of interest statement

PR: None; AG: None; UA: None; RA: consulting fees and research support from Medtronic, Inc.; HC: Donation support from Medtronic Inc., Research support from National Science Foundation (Engineering Research Center for Sensorimotor Neural Engineering); CB: consultant for Boston Scientific, St Jude Medical, NeuroPace, Intelect Medical, Advanced Bionics and Functional Neuromodulation. CB is a shareholder of Intelect Medical. CB has authored intellectual property related to deep brain stimulation; CD: Aspects of work described in this review were supported through DARPA SUBNETS, DARPA/ARO Contract # W911NF-14-2-0043; WE: research funding from: Insightec, Ltd. and Focused Ultrasound Foundation; GG: None; MF: supported from NIH grant K23NS083741 and the Sidney Baer Foundation, listed as an inventor on submitted or issued patents on guiding neurological interventions with fMRI; JG: grant support from the William H. and Ruth Crane Schaefer Endowment; Children's Hospital and Clinics Foundation; the Clark Foundation; and the United States Air Force Office of Scientific Research; RG: consultant for Medtronic, Inc. and St. Jude Medical Corp., receives compensation for these services; WMG: inventor on licensed patents on temporal patterns of deep brain stimulation and owns equity in Deep Brain Innovations, LLC.; JH: Donation support and consulting fees from Medtronic. TH: None; JJ: None; BK: consultant for Medtronic SNT, St Jude Neuromodulation, MRI interventions, NP: fellowship support from Medtronic, paid consultant for Second Sight Medical Products, Inc.; ALang: advisor for Abbvie, Allon Therapeutics, Avanir Pharmaceuticals, Biogen Idec, Boerhinger-Ingelheim, Bristol Myers Squibb, Ceregene, Cipla, Intekrin, Lilly, Medtronic, Merck, Novartis, NeuroPhage Pharmaceuticals, Teva and UCB, honoraria from Medtronic, Teva, UCB, AbbVie, grants from Brain Canada, Canadian Institutes of Health Research, Edmond J Safra Philanthropic Foundation, The Michael J. Fox Foundation, the Ontario Brain Institute, National Parkinson Foundation, Parkinson Society Canada, Physicians Services Incorporated (PSI), Tourette Syndrome Association, and W. Garfield Weston Foundation, publishing royalties from Saunders, Wiley-Blackwell, Johns Hopkins Press, and Cambridge University Press, has served as an expert witness in cases related to the welding industry; CL: None; ALozano: consultant for Aleva, St. Jude Medical, Boston Scientific, Medtronic, Functional Neuromodulation, Inc.; JL: None; AM: consultant for Spinal Modulation Functional Neuromodulation, St Jude Medical, Icahn School of Medicine; Distribution rights to: Enspire, ATI, Cardionomics; Fellowship Support from Medtronic, Research Support from National Institutes of Health.; DM: None; CM: paid consultant for Boston Scientific Neuromodulation and a shareholder in the following companies: Surgical Information Sciences, Inc.; Autonomic Technologies, Inc.; Cardionomic, Inc.; Enspire DBS, Inc.; Neuros Medical, Inc.; AYM: Medtronic: consulting and research support St. Jude: consulting and research support, Boston Scientific: research support; LVM: teaching honorarium from Medtronic, Inc; clinical trial agreement (site PI) with Medtronic, Inc., US WorldMeds LLC, AbbVie, through Quintiles, Avanir Pharmaceuticals Inc.; The Michael J. Fox Foundation, St. Jude Medical Inc.; fellowship grant from Medtronic, Inc.; consortium grant from NIH Research Grant R01 NS40902/P.I. Corcos; RM: None; KGO: None; EO: None; AP: None; AS: None; KGS: paid employee of Boston Scientific Neuromodulation; JS: None; NS: None; AT: research funding from The Michael J. Fox Foundation and Barrow Neurological Foundation, Consultant/Scientific Advisory Board member for Medtronic, Boston Scientific, Teva, St Jude, and Theravance, Royalties from Oxford University Press. MT: consulting fees and research support to my institution from Medtronic, St Jude, and Boston Scientific, and speaker honoraria from Medtronic.; LS: None; CV: None; NV received a Medtronic Fellow Education Scholarship 6/2014-6/2015”; LV: Clinical trial agreement with Medtronic, AbbVie, Avanir Pharmaceuticals, and St. Jude Medical. Teaching honorarium and fellowship grant from Medtronic. Consultant for St. Jude Medical and Depomed; KZ: None; KF: None; MO: consultant for the National Parkinson Foundation; research grants from NIH, NPF, the Michael J. Fox Foundation, the Parkinson Alliance, Smallwood Foundation, the Bachmann-Strauss Foundation, the Tourette Syndrome Association, and the UF Foundation; participated in CME activities on movement disorders sponsored by PeerView, Prime, and by Vanderbilt University. The reviewer FH declared a past co-authorship with author GW and a shared affiliation with authors GW and BK to the handling Editor, who ensured that the process met the standards of a fair and objective review.

## Abbreviations

DBS: deep brain stimulation
FDA: Food and Drug Administration
IIR: investigator-initiated research
IDE: investigational device exemption
NIH: National Institutes of Health
RoR: right of reference
SEEG: stereoencephalography
MRI: magnetic resonance imaging
DTI: diffusion tensor imaging
FA: fractional anisotropy
MCT: modulated circuit tractography
SAS: subtraction activated SPECT
fcMRI: functional connectivity magnetic resonance imaging
TAM: tractography activation models
PD: Parkinson's disease
IPG: implantable pulse generator
MICC: multiple independent current control
HIFU: high intensity focused ultrasound
LFP: local field potential
ECoG: electrocorticography
STN: subthalamic nucleus
ViM: ventralis intermedius
TS: Tourette's syndrome
CM-PF: centromedian parafascicular nucleus of the thalamus
SVM: support vector machine
SC: subcallosal cingulate gyrus
TRD: treatment resistant depression
PFG: prefrontal gyrus
EEG: electroencephalography
LHb: lateral habenula
ECT: electroconvulsive therapy
CSF: cerebrospinal fluid
PTSD: post-traumatic stress disorder
CAPS: clinican-administered PTSD scale
BLn: basolateral nucleus of the amygdala.
